# Peptide-Decorated Degradable Polycarbonate Nanogels for Eliciting Antigen-Specific Immune Responses

**DOI:** 10.3390/ijms242015417

**Published:** 2023-10-21

**Authors:** Judith Stickdorn, Christian Czysch, Carolina Medina-Montano, Lara Stein, Lujuan Xu, Maximilian Scherger, Hansjörg Schild, Stephan Grabbe, Lutz Nuhn

**Affiliations:** 1 Max Planck Institute for Polymer Research, 55128 Mainz, Germany; 2 Department of Dermatology, University Medical Center, Johannes Gutenberg-University Mainz, 55131 Mainz, Germany; 3 Institute of Immunology, University Medical Center, Johannes Gutenberg-University Mainz, 55131 Mainz, Germany; 4 Zhejiang Cancer Hospital, The Key Laboratory of Zhejiang Province for Aptamers and Theranostics, Hangzhou Institute of Medicine (HIM), Chinese Academy of Sciences, Hangzhou 310022, China; 5 Chair of Macromolecular Chemistry, Institute of Functional Materials and Biofabrication, Department of Chemistry and Pharmacy, Julius-Maximilians-Universität Würzburg, 97074 Würzburg, Germany

**Keywords:** aliphatic polycarbonate, post-polymerization modification, peptide antigen, polymer peptide conjugate, nanovaccine

## Abstract

For successful therapeutic interventions in cancer immunotherapy, strong antigen-specific immune responses are required. To this end, immunostimulating cues must be combined with antigens to simultaneously arrive at antigen-presenting cells and initiate cellular immune responses. Recently, imidazoquinolines have shown their vast potential as small molecular Toll-like receptor 7/8 (TLR7/8) agonists for immunostimulation when delivered by nanocarriers. At the same time, peptide antigens are promising antigen candidates but require combination with immune-stimulating adjuvants to boost their immunogenicity and exploit their full potential. Consequently, we herein present biodegradable polycarbonate nanogels as versatile delivery system for adjuvants within the particles’ core as well as for peptide antigens by surface decoration. For that purpose, orthogonally addressable multifunctional polycarbonate block copolymers were synthesized, enabling adjuvant conjugation through reactive ester chemistry and peptide decoration by strain-promoted alkyne-azide cycloaddition (SPAAC). In preparation for SPAAC, CD4^+^-specific peptide sequences of the model protein antigen ovalbumin were equipped with DBCO-moieties by site-selective modification at their *N*-terminal cysteine. With their azide groups exposed on their surface, the adjuvant-loaded nanogels were then efficiently decorated with DBCO-functional CD4^+^-peptides by SPAAC. In vitro evaluation of the adjuvant-loaded peptide-decorated gels then confirmed their strong immunostimulating properties as well as their high biocompatibility. Despite their covalent conjugation, the CD4^+^-peptide-decorated nanogels led to maturation of primary antigen-presenting cells and the downstream priming of CD4^+^-T cells. Subsequently, the peptide-decorated nanogels loaded with TLR7/8 agonist were successfully processed by antigen-presenting cells, enabling potent immune responses for future application in antigen-specific cancer immunotherapy.

## 1. Introduction

The development of vaccines revolutionized the world’s response towards infectious diseases throughout the most recent centuries and is estimated to prevent millions of deaths yearly [1,2]. During the last decades, the scope of possible antigenic moieties that induce an adaptive immune response expanded from inactivated or attenuated pathogens to proteins in subunit vaccines [2]. In recent years, accelerated by the COVID-19 pandemic, even smaller immunogenic cargos such as peptides [3] and mRNAs [4] showed their vast potential for mass application in vaccines [5]. Being readily adaptable, peptide and mRNA-based vaccines can be employed against fast mutating viruses or highly heterogeneous cancer types based on their presently well-established and automated synthesis of distinct epitope sequences [6].

Proteins and peptides generally exhibit reduced immunogenicity compared to attenuated pathogens [7]. Thus, towards an effective therapeutic treatment with these subunit vaccines, not only the proper selection of the antigen but also a precise co-delivery with immune cell-stimulating adjuvants is of importance [8]. In this regard, Toll-like receptor (TLR) agonists that evoke a strong innate and adaptive immune stimulation can be employed. These agonists include nucleic acid adjuvants [9,10] (poly(I:C)-TLR3 and CpG-TLR9) as well as small molecular agonists such as imidazoquinolines (TLR7/8) [11,12,13]. These agonists bind to the TLRs expressed on endosomal membranes of antigen-presenting cells (APCs), e.g., dendritic cells (DCs), leading to their maturation and the upregulation of MHC-I and II expression besides co-stimulatory signaling and the release of pro-inflammatory cytokines that lead to the downstream activation of other immune cells [14]. Based on their capacity to present exogenous antigens to T cells, the activation and maturation of APCs like dendritic cells is a prerequisite for eliciting potent immune reactions [15,16]. This process, called cross-presentation, leads to the induction of antigen-specific cytotoxic CD8^+^ T cells that are presumed crucial in eliminating malignant tumor cells [17]. Apart from CD8^+^ cell epitopes, peptide-based vaccines must also contain epitopes of CD4^+^ T cells to ensure the generation of antigen-specific T helper cells. The resulting interaction of those T helper cells with antigen-specific CD8^+^ T cells is necessary to provoke an effective cellular immunity [14].

Beyond cheaper and faster production when compared to full proteins, peptide antigens also allow sequence-selective tuning for specific (e.g., mutated) epitopes. Especially in targeting patient-specific tumor neoantigens, peptide antigens can be tailored to an individual’s neoantigen repertoire [6,18]. Due to their fully synthetic character, they also improve drug safety by diminishing risks of contaminations from pathogens and toxins [19]. Yet, the absence of inherent immunogenicity has to be taken into account prior to administration. It allows for an improved control over respective downstream immune responses. Depending on the co-administration of potent adjuvants of choice a precise activation of desired immune responses can be achieved and fine-tuned [20].

However, when adjuvants and peptide antigens are applied as such, they distribute systemically and the adjuvants in particular can trigger severe off-target toxicities [21,22,23,24,25]. Nanoparticular delivery allows for a more localized immunostimulation focused to sites of the immune system (e.g., lymph nodes and spleen) [26,27,28]. Thus, nanoparticles can improve the drug’s therapeutic window and safety [29]. In addition to that, nanoparticles can mimic pathogens by presenting multiple antigens on their surface. Therefore, they enable multivalency effects which have been shown to elicit stronger immune responses than the single presentation of antigens and thus ameliorate immunotherapeutic interventions [30,31,32].

For the safe application of nanoparticulate vaccines, it is of great importance that the polymeric materials used are biocompatible [33]. In this regard, aliphatic polycarbonates are a promising material class, even offering a degradable polymer backbone that permits disintegration after therapy [34,35,36]. Polycarbonate’s degradation results in weak acid carbon dioxide and hydrophilic diols, which generally do not exert adverse effects but can get excreted from the body via renal clearance [37]. Moreover, a plethora of functional polycarbonates is readily accessible by polymerizing monomers with various ester or ether side chains [36,38,39]. Most relevant for the generation of functional materials, 5-methyl-5-pentafluorophenyloxycarbonyl-1,3-dioxan-2-one (MTC-PFP), a monomer with a reactive ester side chain, was polymerized via cationic ring-opening polymerization (ROP) [40,41]. After polymerization, these active ester side chains allow for the tuning of material properties via post-polymerization modification with amines [42,43]. Similar to other polymeric architectures that were successfully applied to generate nanocarriers for immunostimulation by the post-polymerization modification approach [44,45,46], reactive ester polycarbonates are suitable materials in this regard [47]. Recently established polycarbonate carriers were not only shown to be readily modifiable, but are also hydrolytically degradable in physiological media, enabling spatio-temporal control over immunostimulation via degradation [48]. Moreover, they allow for the attachment of multiple functionalities for immunodrug delivery such as small molecular adjuvants, dyes, or cross-linkers for enhanced stability. However, for further development towards vaccination, immunostimulatory nanocarriers must be combined with antigens to elicit strong antigen-specific immune responses [28,49,50,51].

**Figure 1:**
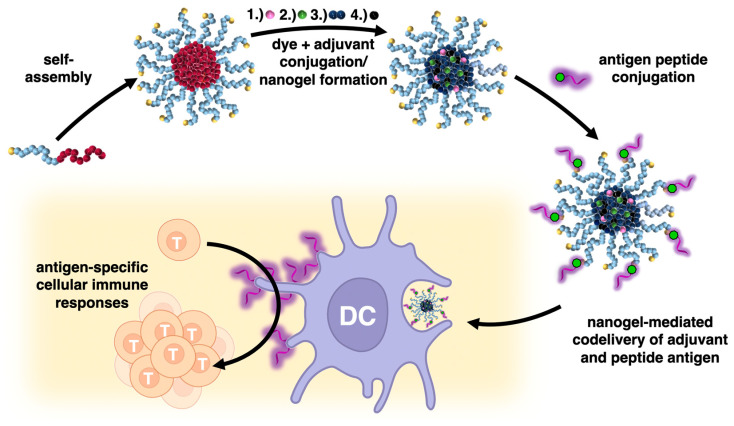
The strategy of covalent co-delivery of small molecular imidazoquinoline-based TLR 7/8 agonists with peptide antigens via polycarbonate block copolymer-derived nanogels. Core-functionalization through sequential reactive ester chemistry conversion is combined with biorthogonal strain-promoted alkyne-azide cycloaddition (SPAAC) click chemistry on the surface of the nanogel to generate both adjuvant- and antigen-loaded biodegradable nanogels that efficiently co-deliver both entities to antigen-presenting dendritic cells and trigger an antigen-specific T-cell proliferation.

For that purpose, we expanded the strategy of polycarbonate nanogels derived from MTC-PFP by orthogonally addressable azide click groups at the surface of the nanogels, which allow for a covalent conjugation of DBCO-modified peptide antigens via strain-promoted alkyne-azide cycloaddition (SPAAC). Both the antigen- and adjuvant-loaded biodegradable nanogels can effectively co-deliver both entities to antigen-presenting cells for eliciting antigen-specific immune responses, thus serving as promising customizable nanovaccines (Figure 1).

## 2. Results and Discussion

**Figure 2:**
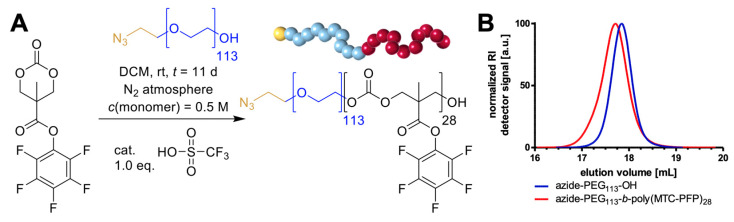
Polycarbonate block copolymer synthesis via organocatalyzed ring-opening polymerization (ROP) of 5 methyl-5-pentafluorophenyloxycarbonyl-1,3-dioxan-2-one (MTC-PFP) initiated by azide functionalized polyethylene glycol (azide-PEG_113-_OH). (**A**) Cationic ROP of MTC-PFP catalyzed by trifluoromethanesulfonic acid (TFMSA) affording azide-PEG_113_-*b*-poly(MTC-PFP)_28_ block copolymers. (**B**) Size exclusion chromatography (SEC) elugram recorded by a refractive index (RI) detector of azide-PEG_113_-OH (blue, *Ð* = 1.10) and azide-PEG_113_-*b*-poly(MTC-PFP)_28_ (red, *Ð* = 1.18), showing narrowly distributed homo and block copolymers.

We opted for the polycarbonate nanogel platform with the aim of combining them with peptide antigens via functionalization of the particular surface. For that purpose, polymers containing reactive moieties were synthesized and subsequently assembled to form functionalizable nanocarriers. Extending the nanogel platform for covalent attachments to the nanoparticular surface, we opted for azide-functional block copolymers that enable functionalization through orthogonally addressable click chemistry. Such block copolymers can be synthesized using functional macroinitiators with a clickable end group such as α-azido-ω-hydroxy-terminated polyethylene glycol (azide-PEG_113_-OH). Polymerizing a second polymer block from this macroinitiator should enable the self-assembly to form polymeric micelles and subsequently convert them into nanogels via post-polymerization modification. Therefore, the six-membered active ester monomer MTC-PFP was polymerized by cationic ROP initiated from azide-PEG_113_-OH (Figure 2A). Ensuring ideal reaction conditions for a controlled polymerization, reagents were thoroughly dried beforehand, and the reaction was conducted in a nitrogen-purged glove box. By these means, narrowly distributed block copolymers were obtained as analyzed using size exclusion chromatography (Figure 2B, *Ð* = 1.18 and Figure S1). Further characterization data acquired with NMR spectroscopy and mass spectrometry confirmed the controlled synthesis of the block copolymer azide-PEG_113_-*b*-poly(MTC-PFP)_28_ (Figures S2–S4).

**Figure 3:**
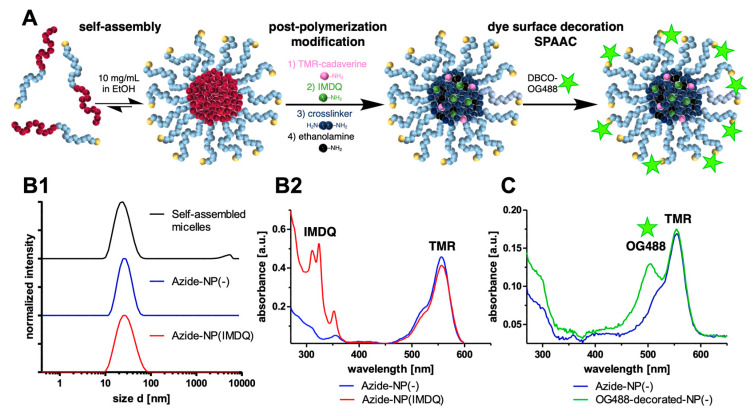
Preparation of azide-functional polycarbonate nanogels from azide-PEG_113_-b-poly(MTC-PFP)_28_ by post-polymerization modification and their surface-modification by strain-promoted alkyne-azide cycloaddition (SPAAC). (**A**) Nanogel fabrication via self-assembly of reactive precursor polymers in ethanol, forming azide-functional polymeric micelles. Post-polymerization modification by attachment of amine-functional molecules such as tetramethylrhodamine (TMR) cadaverine fluorescent dye, TLR7/8 agonist IMDQ and hydrophilic crosslinker 1,8-diamino-3,6-dioxaoctane. By these means, drug-loaded nanogels (azide-NP(IMDQ)) and non-drug-loaded nanogels (azide-NP(-)) were prepared. Surface functionalization of the azide-functional nanogels and the accessibility of the azide moieties on the surface was then demonstrated by attaching the DBCO-modified fluorescent dye Oregon Green 488 (OG488—green star). (**B**) Characterization data of azide-functional nanogels. (**B1**) Dynamic light scattering (DLS) measurements confirming the uniform size of nanogels (azide-NP(-), blue) and IMDQ-loaded nanogels (azide-NP(IMDQ), red), comparable to the initially self-assembled micelles (black). (**B2**) UV–Vis spectra of the nanogels showing both the TMR absorption at ~550 nm and the IMDQ absorption for azide-NP(IMDQ) at ~321 nm. (**C**) UV–Vis spectra of the surface functionalization of azide-NP(-) with DBCO-modified fluorescent dye OG488. After SPAAC and thorough removal of excess dye, the additional absorbance of OG488 at ~488 nm demonstrated successful surface modification to yield OG488-decorated-NP(-) (green). A control sample of azide-NP(-) treated with OG488 cadaverine showed no conjugation to the nanogel and quantitative removal during spin-filtration workup (blue line).

These active ester precursor polymers were then employed in the fabrication of azide-functional nanogels (Figure 3). First, polymers were assembled into micelles (Figure 3(B1), top) to form narrowly distributed nanoparticles (z-ave. = 23.6 nm; PDI = 0.20). These assemblies were then functionalized in a post-polymerization modification approach. Therefore, amine functional molecules were covalently attached to the nanogels by amidation reactions. Starting with the attachment of amine functional tetramethylrhodamine (TMR) cadaverine and TLR7/8 agonist IMDQ, small molecular cargos were loaded that later act as sensors within biological systems and boost strong immune responses, respectively. The micelles were core-crosslinked with the hydrophilic bisamine 1,8-diamino-3,6-dioxaoctane, forming fully hydrophilic nanogels. Ensuring the complete removal of the remaining reactive ester sidechains within the nanogels, an excess of ethanolamine was added. By altering the attached amines, both drug-loaded and non-drug-loaded nanogels were prepared. Byproducts such as pentafluorophenol as well as other non-bound drugs or dyes were then removed by dialysis. The resulting nanogels were again analyzed by DLS (Figure 3(B1)) and showed sizes of 25.3 nm (z-ave. and PDI of 0.15) for IMDQ-loaded nanogels (azide-NP(IMDQ), blue line) and of 26.4 nm (z-ave. and PDI of 0.08, red line) for non-loaded nanogels (azide-NP(-)). Note that both nanogels provided similar sizes as their initially self-assembled micelles. Additionally, the nanogels revealed a gradual disintegration behavior at physiological conditions (PBS, pH 7.4, 37 °C) over a timespan of several days, forming small molecular degradation products (Figure S5). These properties underline the polycarbonate-based hydrolytic degradation feature favorable for biodegradation and transient immunodrug delivery purposes [48]. Additionally, covalent IMDQ drug-load was determined via UV–Vis measurement (Figure 3(B2)), and applying an external IMDQ calibration curve afforded a drug-load of 4.6 wt% (Figure S6).

For the following preparation of peptide-decorated nanogels using the anticipated click approach, it had to be ensured that the nanocarriers’ azide-groups were accessible and still able to react with their alkyne counterparts during SPAAC. In this regard, the attachment of a DBCO-modified fluorescent dye (OG488) to the surface of the carriers was attempted. After work-up by excessive spin filtration for the removal of potentially unreacted dye, an additional UV-absorption at 488 nm was found (Figure 3C, green line). Contrarily, the control reaction where an OG488 cadaverine dye was used instead showed that the dye was not conjugated to the nanogels and quantitively removed during spin-filtration workup (Figure 3C, blue line and Figure S7). Therefore, it was proven that azide-groups are indeed accessible for click reaction, enabling the efficient surface decoration of nanogels.

Proven reactive towards DBCO-displaying molecules, we proceeded with the conjugation of antigenic peptides to the azide-functional nanogels. An antigenic peptide sequence was selected based on the CD4^+^ T cell epitope of the model protein ovalbumin (OVA) [52], which is widely used to evaluate vaccine delivery systems for cancer immunotherapy [50,53,54]. We previously demonstrated that the attachment of the full protein to the surface of IMDQ-loaded methacrylate-based nanogels is able to trigger profound cellular and humoral immune responses [50]. Here, we wanted to expand the strategy towards the application of antigenic peptide sequences that can specifically initiate CD4^+^-T cell responses exclusively. To ensure the co-delivery of peptides and adjuvants into the same immune cell populations and an improved immune response, both compounds should be combined into the same nanocarrier [53,55,56,57].

**Figure 4:**
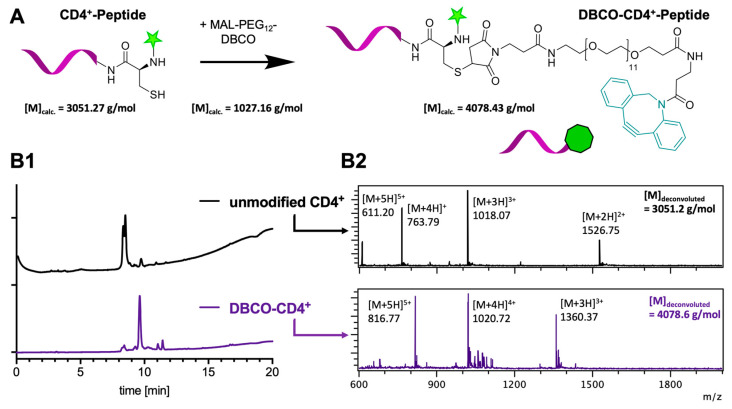
Ovalbumin-derived CD4^+^-peptide antigen modification. (**A**) Reaction of the peptide’s *N*-terminal cysteine thiol moieties with maleimide-functional DBCO (MAL-PEG_12_-DBCO) by Michael-addition. For fluorescence tracing, the peptides are covalently equipped with 5-carboxyfluorescein (5-FAM) dye (green star) at their *N*-terminus. (**B**) Characterization results by LCMS. (**B1**) HPLC trace of DBCO-conjugated CD4^+^-peptide antigen detected by UV–Vis at 254 nm in comparison to unmodified CD4^+^-peptide before Michael-addition of MAL-PEG_12_-DBCO. (**B2**) ESI-MS data of the respective ionized species for unmodified and DBCO-conjugated CD4^+^-peptide antigen. After deconvolution an increase in molecular weight of 1027.4 g/mol was found for the modified CD4^+^-peptide antigen which corresponds to the molecular weight of MAL-PEG_12_-DBCO.

For this purpose, the selected peptide sequence had to be modified with a DBCO-functionality for the subsequent SPAAC conjugation to nanogels. Hence, we exploited the *N*-terminal cysteine for a site-selective modification with DBCO-PEG_12_-maleimide (Figure 4A). For monitoring the delivery process of the peptide by the nanogel to the antigen-presenting cells, we used a peptide sequence that was further equipped with covalently attached 5-carboxyfluorescein (5-FAM) at its *N*-terminus (5-FAM-CSSAESLKISQAVHAAHAEINEAGR). Successful DBCO-modification was determined by LC-MS (Figure 4(B1,B2) and showed nearly complete conversion by a quantitative shift in the HPLC trace compared to the unmodified peptide (Figure 4(B1)). In addition, expected molecular weights of DBCO-modified peptide conjugates were found by ESI-MS, while traces of unmodified peptide residues remained elusive (Figure 4(B2), Figures S8 and S9). Based on these results, the DBCO-modified CD4^+^ peptide was directly used without further purification for the SPAAC conjugation to the azide-nanogels.

**Figure 5:**
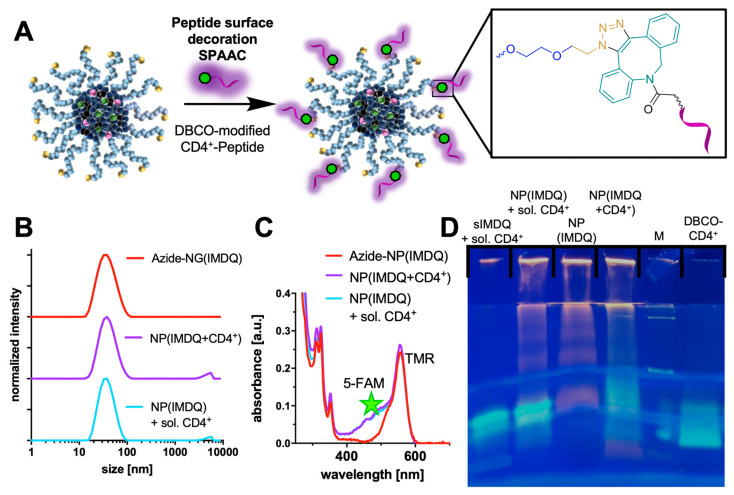
Surface decoration of adjuvant-loaded polycarbonate nanogels. (**A**) Conjugation of CD4^+^-peptide antigens to the nanogel surface by SPAAC. Magnification of the triazol product (right) that covalently links the peptides to the nanogel surface. (**B**) DLS measurements of azide-NP(IMDQ) and NP(IMDQ+CD4^+^) after SPAAC and control (NP(IMDQ) + sol. CD4^+^). (**C**) UV–Vis measurements of NPs showing additional UV absorption at 488 nm of 5-FAM after addition of the CD4^+^-peptide solution. (**D**) SDS-PAGE proving the covalent attachment of DBCO-CD4^+^ to the nanogel surface (NP(IMDQ+CD4^+^).

Consequently, the covalent attachment of peptides to nanogels was pursued by mixing the DBCO-modified CD4^+^-peptide antigens with the IMDQ-loaded azide-functional nanocarrier in water (supplemented with hydrochloric acid at pH 4 to avoid premature hydrolytic nanogel degradation) (Figure 5A). SPAAC conjugation was performed in a molar ratio of 1:9 (DBCO to azide) and analyzed by sodium dodecyl sulfate–polyacrylamide gel electrophoresis (SDS-PAGE). Fluorescent labeling of both peptide (5-FAM) and nanogel (TMR) allowed for a direct optical visualization of successful conjugation on the polyacrylamide gel upon illumination (Figure 5C for UV–Vis absorbance spectra). For the conjugation of the modified CD4^+^-peptide antigen, the band of the free DBCO-modified CD4^+^-peptide disappeared after incubation of the peptides with nanogels (Figure 5D). In the case of mixing the non-DBCO modified soluble CD4^+^-peptide with the nanogel, no change in band intensity was observable for the free peptide. Furthermore, the successful conjugation of the CD4^+^-peptide was confirmed by a yellowish trace in the sample well and at the intersection between stacking and separating gel, where the nanogel NP(IMDQ) or the control sample (NP(IMDQ)+sol. CD4^+^) provided a red-orange color(Figure 5D).

During subsequent characterization of the nanogel formulations by DLS measurements, uniform sizes without major differences between the samples were recorded (Figure 5B). IMDQ-loaded nanogels showed sizes of 34.5 nm (z. ave. size and PDI of 0.15) and CD4^+^-peptide antigen-decorated nanogels of 38.7 nm nm (z. ave. size and PDI of 0.23). The mixture of IMDQ-loaded nanogels and soluble CD4^+^-peptide antigens exhibited a size of 36.3 nm (z. ave. size and PDI of 0.18). Hence, attachment of the small peptides into the nanogel corona did not alter the nanogels’ dimensions significantly. Additionally, atomic force microscopy (AFM) images confirmed the spherical shape of the peptide-decorated nanogels (Figure S10). Further characterization was performed by UV–Vis measurements and confirmed similar IMDQ-loading as well as dye-functionalization for soluble or conjugated CD4^+^-peptide antigens to nanogels for subsequent in vitro analyses (Figure 5C).

Having established the fabrication of adjuvant-loaded nanogels with a peptide-decorated surface, we next evaluated the biological behavior of the carrier system. To this end, first in vitro tests were conducted on RAW Blue macrophages to evaluate the nanogels’ immunostimulation and biocompatibility [58]. These experiments confirmed that IMDQ-loaded nanogels are able to effectively stimulate macrophages at concentrations in the µM range (Figure S11). Moreover, they do not exert negative effects on cell viability as investigated by MTT assays (for particle concentrations of up to 0.061 mg/mL).

**Figure 6:**
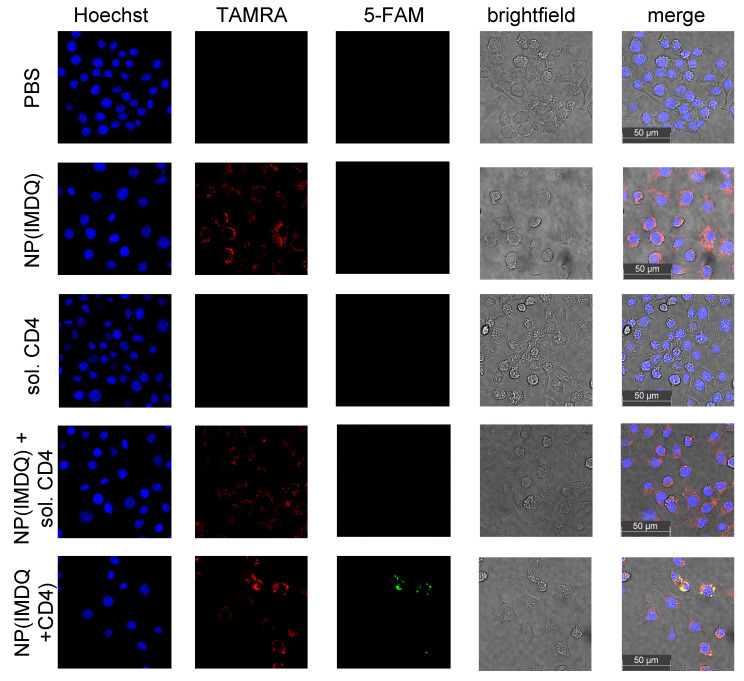
Confocal microscopy on RAW macrophages showing the internalization of nanogels by TMR fluorescence (red) and synchronously the co-uptake of CD4^+^-peptides by 5-FAM fluorescence (green) only when covalently attached to the nanogel. Nuclei were stained with Hoechst dye (blue) and bright-field images (grey) were used for visualizing cellular morphologies.

Next, the co-delivery potential of peptide-decorated particles was investigated on this cell line by confocal microscopy images according to the dual labeling of nanogel (TMR) and antigen peptide (5-FAM). As shown in Figure 6, nanogels are generally well internalized by the RAW macrophage cells and provide a signal in the TMR-channel. However, detection of 5-FAM fluorescence related to the CD4^+^-peptide antigen can only be observed when it is covalently attached to the nanogel. Non-conjugated CD4^+^-peptide alone or mixed with the nanogel cannot become internalized by the cells. Only after SPAAC-mediated covalent conjugation to the nanogel surface can a co-localization of the 5-FAM fluorescence of the CD4^+^-peptide with TMR fluorescence of the nanogels be found (Figure 6 and Figure S12).

**Figure 7:**
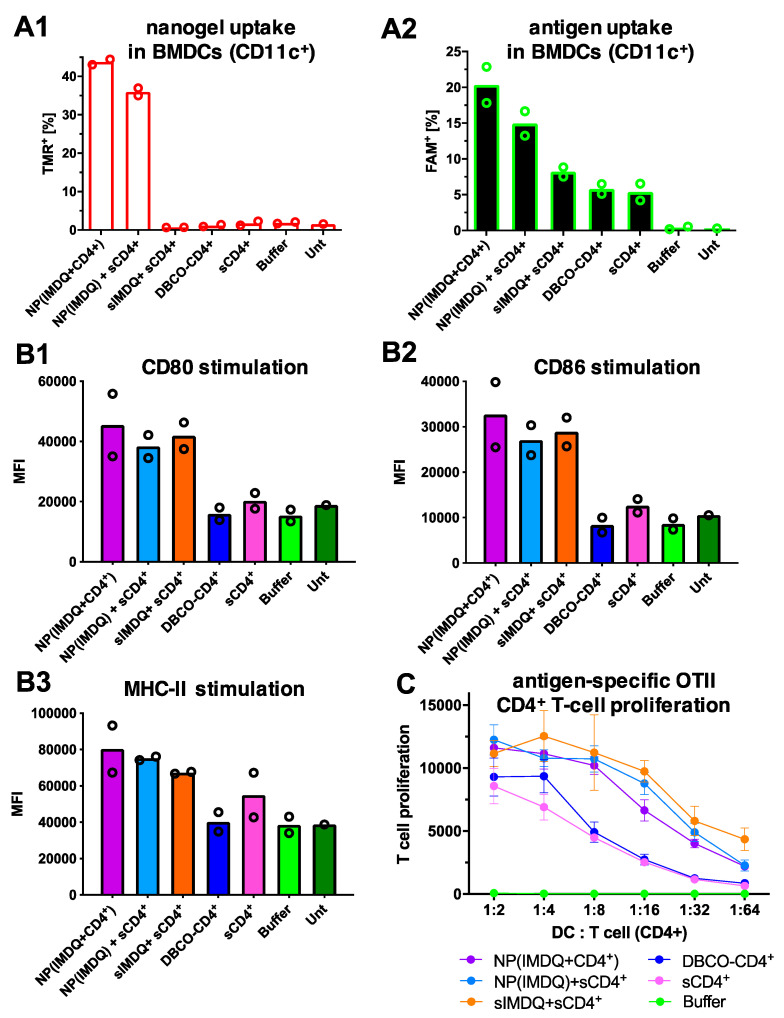
In vitro immune stimulatory evaluation of CD4^+^-peptide antigen-decorated nanogels NP(IMDQ+CD4^+^) on bone marrow-derived dendritic cells (BMDCs) as two biological replicates. (**A**) Uptake of CD4^+^-peptide antigen-decorated nanogels into BMDCs determined by flow cytometry. (**A1**) Nanogel uptake by TMR fluorescence. (**A2**) CD4^+^-peptide antigen uptake by FAM fluorescence. (**B**) The maturation of BMDCs determined by flow cytometry. An upregulation of maturation markers can be found for those cells that were treated with samples containing the TLR7/8 agonist IMDQ (**B1**) Stimulation of maturation marker CD-80. (**B2**) Stimulation of maturation marker CD-86. (**B3**) Stimulation of maturation marker MHC-II. (**C**) Dendritic cells incubated with adjuvant and CD4^+^-peptide antigen-containing samples efficiently stimulate CD4^+^-peptide antigen-specific T cells. In a co-culture model experiment of BMDCs with OTII cells, a strong proliferation of the CD4^+^-peptide antigen-specific T cells was found even at a low DC:T cell ratio. Similar antigen-specific proliferations were found when both components (IMDQ adjuvant and CD4^+^-peptide antigen) were covalently ligated and co-delivered by the polycarbonate nanogels.

Since antigens need to be processed and presented by antigen-presenting cells (APCs) for the subsequent activation and priming of T cells, further experiments were performed on primary antigen-presenting cells (APCs). Incubation of bone marrow-derived dendritic cells (BMDCs) with peptide-decorated nanogels followed by flow cytometric quantification provided similar uptake behavior as observed before on RAW Blue macrophages (Figure 6). While samples containing nanogels provided signals in the TMR channel (Figure 7(A1)), superior signals for the FAM-labeled CD4^+^-peptide antigens were preferentially found when they were covalently attached to the nanogel surface as NP(IMDQ+CD4^+^) (Figure 7(A2)). These observations confirm again the successful co-delivery properties for polycarbonate nanogels for promoting the cellular internalization of peptide-based antigens after covalent conjugation to the nanogel surface.

More significantly, the antigen- and adjuvant-loaded nanogels subsequently stimulate the BMDCs and efficiently promote their maturation. For those BMDC samples, the expression of the typical maturation markers CD80 (Figure 7(B1)), CD86 (Figure 7(B2)), and MHC-II (Figure 7(B3)) were upregulated when they were exposed to soluble or nanogel-bound IMDQ. Since maturated DCs are a prerequisite for downstream activation of other immune cells, these results motivated us to further investigate the effective peptide antigen presentation on APCs (Figure 7C). Based on this, the downstream activation of antigen-specific CD4^+^ T cells can be monitored via co-culturing and proliferation. To these means, OVA-specific OT-2 cells, corresponding to OVA-specific CD4^+^ T cells, were co-cultured with maturated BMDCs, and T cell proliferation was then analyzed via incorporation of radioactive ^3^H-thymidine. Interestingly, these antigen-specific CD4^+^ T cells became exclusively stimulated and proliferated most effectively when both adjuvant and peptides were presented on the same particle or co-administered (Figure 7C). Neither the covalent conjugation of the peptide antigen nor the small molecule TLR7/8 adjuvant to the nanogel impacted their biological activity. These experiments therefore exemplify the unique immunostimulation properties of the adjuvant-loaded peptide-decorated nanogels and their ability to elicit antigen-specific immune responses.

## 3. Materials and Methods

### 3.1. Materials

Solvents and reagents were purchased from commercial suppliers and used without further purification unless otherwise mentioned. Trifluoromethanesulfonic acid, benzene (dry), methylene chloride (dry), diethyl ether, dimethyl sulfoxide (DMSO), acetonitrile (ACN, HPLC grade), ethanol amine, 3,6-dioxa-1,8-diaminooctane, DBCO-PEG_12_-maleimide and phosphate-buffered saline (PBS) tablets were purchased from Sigma-Aldrich. Azido poly (ethylene glycol) methyl ether (azide-PEG_113_-OH) was purchased from Rapp Polymere, 5(6)-tetramethyl rhodamine (TMR) cadaverine was purchased from Biotium, and the peptide sequence was obtained from GenScript. 1-(4-(aminomethyl)-benzyl)-2-butyl-1H-imidazo[4,5-*c*]quinolin-4-amine (IMDQ) was synthesized as reported here [59,60,61] and 5-methyl-5-pentafluorophenyloxycarbonyl-1,3-dioxan-2-one (MTC-PFP) was synthesized as reported here [48]. Millipore water was prepared using a MILLI-Q R Reference A+ system at a resistivity of 18.2 MΩ·cm and total organic carbon of <5 ppm. Dialysis was performed using Spectra/Por^®^ 7 dialysis membranes with a molecular weight cutoff of 1000 g mol^−1^, and Amicon spin filters were used with a molecular weight cut-off (MWCO) of 10,000 g mol^−1^ obtained from Merck Millipore.

### 3.2. Instrumentation

^1^H and ^19^F NMR spectra were recorded on a Bruker Avance 400 MHz spectrometer (Bruker, Billerica, MA, USA). Samples were prepared using deuterated solvents obtained from Sigma-Aldrich (St. Louis, MO, USA), and the resulting spectra were analyzed using MestReNova 14.2.0 by Mestrelab Research.

Analytical size exclusion chromatography (SEC) was carried out on a SECcurity^2^instrument from PSS Polymer Standards Service GmbH with 1,1,1,3,3,3-hexafluoroisopropanol (HFIP) as eluent, containing 3 g/L potassium trifluoroacetate. The system was equipped with a SECcurity^2^isocratic pump, a degasser, an auto sampler, and a column thermostat. The flow rate was set at 0.8 mL/min, and the column was equilibrated at 40 °C. The column material was composed of modified silica gel (PFG columns, particle size: 7 μm, porosity: 100 Å + 1000 Å) from PSS. For polymer detection, a UV detector at a wavelength of λ = 230 nm and a RI detector were employed. Furthermore, molecular weights were determined via calibration with PMMA (PSS). Evaluation of the elution diagram was conducted with PSS WinGPC softwareUniChrom 8.4.

Dynamic light scattering measurements were performed on a Malvern Z Nano instrument containing a He-Ne-Laser (λ = 632.8 nm). Measurements were performed in triplicates at a detection angle of 173°. For sample analysis, ZetaSizer Software 7.12 was applied. 

For atomic force microscopy imaging, nanogels were diluted to 50 mg/L, drop-casted (5 µL) onto a freshly cleaved mica substrate (circular, 15 mm). AFM imaging measurements were performed using a Bruker Dimension FastScan Bio^®^atomic force microscope in Peak Force mode. Samples were scanned with scan rates of 0.994–1.41 Hz, and AFM probes with a nominal spring constant of 0.25 N/m (FastScan-D, Bruker) were employed. Data was processed using NanoScope Analysis 1.8.

UV–Vis spectra were recorded on a Thermo Scientific™ NanoDrop™ 2000c spectrophotometer using Brand UV micro cuvettes.

Fluorescence spectroscopy measurements as well as multi-well UV–Vis absorbance spectra for RAW-blue and MTT assays were conducted on a Spark 20M Multimode Microplate Reader from TecanTrading (TECAN plate reader, Tecan, Männedorf, Switzerland).

Matrix-assisted laser desorption ionization time of flight mass spectrometry (MALDI-ToF) measurements were carried out on a rapifleXTM MALDI-TOF/TOF mass spectrometer from Bruker Daltonik GmbH. The instrument is equipped with a scanning smartbeam 10 kHz Nd:YAG laser at a wavelength of 355 nm and a 10 bit 5 GHz digitizer. The acceleration voltage was set to 20 kV, and the mass spectra were recorded in positive ion mode. Calibration was done with polyethylene glycol standards from PSS. Mass spectrometry data were analyzed using mMass.

High performance liquid chromatography coupled with mass spectrometry (LCMS) measurements were performed on a Shimadzu LCMS2020 with a Kinetex 2.6 μm EVO C18 100 Å LC 50 ×2.1 mm column, an electrospray ionization source, and an SPD-20A UV–Vis detector. Millipore water and ACN supplemented with 0.1% formic acid were used as eluents for all the measurements, with gradients running from 5% ACN/95% water to 100% ACN at 16 min, followed by 5% ACN/95% water for a 20 min equilibration prior to the next measurements. Data were processed with LabSolutions provided by Shimadzu.

Sodium dodecyl sulfate polyacrylamide gel electrophoresis (SDS-PAGE) was performed with 15% polyacrylamide separation gels and 5% polyacrylamide loading gels using the Mini-PROTEAN Tetra Cell from Bio-Rad. Samples were prepared by mixing the corresponding volume of 3 μg peptide with 4× reducing Laemmli sample buffer before loading. For molecular weight comparisons, the NovexTM Sharp Pre-Stained Protein Standard was used. Gel electrophoresis was performed for 55 min at 160 mV. Gel images were obtained after excitation with a UV lamp.

### 3.3. Block Copolymer Synthesis of Azide-PEG_113_-b-Poly(MTC-PFP)

The synthesis of azide-PEG_113_-*b*-poly(MTC-PFP) was conducted following a preparation modified from previous block copolymer synthesis for mPEG_113_-*b*-poly(MTC-PFP) [41,48]. 

An amount of 0.200 g (0.040 mmol, 1.0 eq.) of azido functional polyethylene glycol (azide-PEG_113_-OH) and 0.522 g (1.600 mmol, 40.0 eq.) MTC-PFP were dried via azeotropic distillation using 3 mL benzene under reduced pressure in two separate Schlenk tubes. After being transferred into a nitrogen purged glovebox, 1.6 mL of dry DCM was added to each tube. The solubilized azide-PEG_113_-OH was then added to MTC-PFP (final monomer concentration of 0.5 m). For the initiation of cationic ROP, 3.52 μL (0.006 g, 0.040 mmol, 1.0 eq.) trifluoromethanesulfonic acid (TFMSA) was pipetted to the polymerization mixture and stirred for 11 d at room temperature. Monomer conversion was checked regularly by NMR measurements, and the reaction was terminated at a conversion of 86.8% via precipitation into cold diethyl ether. Centrifugation (4500 rpm, 10 min, 4 °C) then yielded the block copolymer as a colorless solid. After two additional precipitation steps in an analogous manner, the polymer was dried under reduced pressure (0.402 g, 62% yield by mass when using the conversion of 86.8%).

Hexafluoroisopropanol (HFIP) SEC calibrated by a PMMA standard:

Azide-PEG_113_-OH: *M_n_* = 3.07·10^4^ g/mol *M_w_* = 3.37·10^4^ g/mol, *Ð* = 1.10

Azide-PEG_113_-*b*-poly(MTC-PFP)_28_: *M_N_* = 3.60·10^4^ g/mol *M_w_* = 4.24·10^4^ g/mol, *Ð* = 1.18

^1^H NMR (300 MHz, CDCl_3_, 25 °C) δ = 4.45 (s, 4 H; *CH_2_* MTC-PFP), 3.64 (s, 4 H; O*CH*_2_*CH*_2_ PEG), 1.49 (s, 3 H; *CH_3_* MTC-PFP).

^19^F NMR (282 MHz, CDCl_3_, 25 °C) δ = −153.1 (2 F; *m*-F), −157.1 (1 F; *p*-F), −162.1 (2 F; *o*-F).

### 3.4. Adjuvant-Loaded Azide-Functional Nanogels

For nanogel preparation with TMR or IMDQ, azide-PEG_113_-*b*-poly(MTC-PFP)_28_ block copolymers were dispersed at 10 mg/mL in ethanol (2% water content by volume) via ultrasonication (20 min at ~35 °C). The size of the micellar assembly was then determined by DLS, showing narrowly distributed particles with z-average sizes of 23.6 nm and a PDI of 0.20. An amount of 1 mL of the particle solution was then transferred to each glass vial. The vessels were equipped with a stir bar and placed on a magnetic stirring plate to ensure thorough mixing during particle functionalization. Amine reactants were added consecutively, and each was allowed to react for 4 min for dye attachment, 10 min IMDQ attachment, and 10 min for core-crosslinking.

Azide-NP(-): TMR Cadaverine 17.2 µL (5 mg/mL in EtOH, 0.086 mg, 0.17 µmol), 0.5 eq. 3,6-dioxa-1,8-diaminooctane 1.44 µL (1.44 mg, 23.7 µmol), and 1.05 eq. Triethylamine 2.87 µL (2.09 mg, 20.7 µmol).

Azide-NP(IMDQ): TMR Cadaverine 17.2 µL (5 mg/mL in EtOH, 0.086 mg, 0.17 µmol), IMDQ 58.0 µL (10 mg/mL in EtOH, 0.58 mg, 1.61 µmol), 1.30 eq. Triethylamine 3.55 µL (2.59 mg, 25.6 µmol), and 0.459 eq. 3,6-dioxa-1,8-diaminooctane 1.32 µL (1.34 mg, 9.05 µmol).

Finally, 1.44 µL of ethanolamine (1.44 mg, 23.7 µmol, 1.2 eq.) was added to each reaction in order to quench any remaining PFP-esters and stirred for further 10 min.

To terminate both reactions, they were added to 4 mL of hydrochloric acid (c = 1 mol/L). The byproducts were removed via dialysis in a tube with a 1 kDa cutoff against 1 L of diluted hydrochloric acid (pH = 4) for about ~24 h. The dialysis medium was exchanged frequently. The nanogels were finally obtained as a red solution and filtered using a 0.2 µm syringe filter. 

An aliquot was freeze-dried to determine the nanogel content of the solution (6.6 mg of azide-NP(-), and 4.8 mg of azide-NP(IMDQ) were obtained from 10 mg of the precursor block copolymer).

### 3.5. SPAAC of Azide-Functional Nanogels with DBCO-Modified Oregon Green 488

Dibenzocycloazaoctyne (DBCO)-modified Oregon Green 488 was prepared by a previously described preparation method [50]. To test the accessibility and reactivity of the azide-groups on the nanogels’ surface, 670 µL nanogel solution in pH 4 HCl (0.5 mg, c = 0.75 mg/mL, 11.079 g/mol per azide group, 45.1 nmol, 1.0 eq.) was mixed with 29.8 µL of DBCO-modified Oregon Green 488 (0.0745 mg, c = 2.5 mg/mL in DMSO, 812.85 g/mol per DBCO group, 91.7 nmol, 2.0 eq.). As a control, a second mixture was prepared using azide nanogels mixed with Oregon Green 488 cadaverine. Lacking DBCO moieties, this dye would not react with the azide-functional nanogels and would be fully removed during work-up. After 4 days of incubation, excess Orgon Green 488 (OG488) dye was removed using spin filters with a molecular weight cut-off (MWCO) of 10,000 and centrifugation at 4000× *g*. Samples were washed repeatedly until the filtrate did not show any detectable UV–Vis absorption. Finally, particle solutions were filled up to 100 µL, and all samples were analyzed again via UV–Vis on a TECAN plate reader.

### 3.6. DBCO-Peptide Modification by Site-Selective Modification of Cysteine

The peptide sequences based on the CD4^+^ (MHC-II) class epitope of ovalbumin (OVA) [52] CSSAESLKISQAVHAAHAEINEAGR was selected and purchased with *N*-terminal fluorescent labeling of 5-FAM-Ahx (5-Carboxyfluorescein) and a *N*-terminal cysteine for further modification. An amount of 150 µg of CD4^+^ epitope peptide (47.4 nmol, 1.0 eq.) was dissolved at 1 mg/mL in a 1:1 mixture of acetonitrile and phosphate buffer (pH 7.4). DBCO-PEG_12_-maleimide (40 mg/mL in DMSO, 1.0 μL, 37.9 nmol, 0.8 eq.) was added, and the mixture was incubated overnight at room temperature. After analysis by LCMS and HPLC showing complete peptide modification, the mixture was directly used for SPAAC conjugation with the azide-nanogels.

LCMS: DBCO-PEG_12_-CD4^+^ peptide: *m*/*z* found: 1020.7 [M+4H]^4+^, *m*/*z* calculated: 1021.1 [M+4H]^4+^.

### 3.7. SPAAC of DBCO-Modified Peptides and Azide-Functional Nanogels

For the generation of CD4^+^ epitope-decorated nanogels, azide-functional nanogels were concentrated to 5 mg/mL in dissolved hydrochloric acid (pH 4) using a vacuum concentrator. Then, 60 μL of the nanogel solution (5 mg/mL, 27 nmol azide, 9.0 eq.) was added to a 9.6 μL DBCO-modified CD4^+^ epitope (1.28 mg/mL, 3 nmol, 1.0 eq.), and the mixture was filled to a total volume of 100 μL with sterile water/hydrochloric acid (pH 4). Similar to that, mixtures of nanogels and non-conjugating soluble peptides were prepared with an unmodified CD4^+^ epitope (1 mg/mL, 9.5 μL, 3 nmol). All formulations were incubated at 4 °C for 48 h before analysis by SDS-PAGE, UV–Vis, and DLS measurements.

### 3.8. IMDQ-Loaded Azide-Functionalized Nanogel TLR Activity on RAW-Blue Macrophage Reporter Cell Line

RAW Blue macrophages can be used as TLR reporter assay because of their downstream activation of AP-1/NF NF-κB and subsequent secretion of SEAP (secreted embryonic alkaline phosphatase). They were purchased from InvivoGen, and the TLR report assay was conducted as recommended by the supplier. Cells were seeded into 96-well plates (90,000 cells/well in 180 µL culture medium) and incubated with 20 µL of the serial dilutions of equivalent IMDQ concentrations. After ~20 h of incubation, 50 µL of the supernatant was collected, added to 150 µL of Quanti Blue solution (InvivoGen, San Diego, CA, USA), and analyzed for the secretion of SEAP. After incubation for 2 h at 37 °C, the wells were read out for their absorbance at 620 nm using a plate reader. All experiments were performed in quadruplicate.

### 3.9. Influence of the IMDQ-Loaded Azide-Functionalized Nanogels on the Metabolic Activity of the Stimulated Raw Blue Macrophages by MTT Assay

To simultaneously analyze the metabolic activity of the cell as an indicator of toxicity and cell proliferation, the previously stimulated cells were incubated with 30 µL of 2 mg/mL 3-(4,5-dimethylthiazol-2-yl)-2,5-diphenyltetrazolium bromide solution in PBS. After 3 h of incubation, the formed formazan crystals were dissolved by the addition of 100 µL of 10% *m*/*v* SDS/0.01 M HCl and incubated overnight at 37 °C. Absorbance was measured at 590 nm using a plate reader. All experiments were performed in quadruplicate.

### 3.10. Confocal Microscopy Studies of Raw Blue Macrophages Stimulated with IMDQ-Loaded and Peptide-Decorated Nanogels

Raw Blue macrophage cells (50,000 cells/well in 180 µL) were seeded in an eight-well polystyrene-chambered cover glass (Laboratory-Tek, Nalge Nunc International, Penfield, NY, USA). Respective amounts of TMR-labeled nanogels and FAM-labeled CD4+ peptides (as covalent conjugates, alone or as mixtures) were added (20 µL solution) and incubated overnight. The medium was removed, and the cells were washed with PBS three times. The cells were fixed with 200 µL of a 4% paraformaldehyde solution for 30 min at 37 °C. Afterwards, the cells were washed again with PBS three times, and the cell nuclei were stained using a 4′,6-diamidino-2-phenylindole (DAPI) solution (80 µg/mL in PBS) for 10 min at 37 °C. Finally, the samples were again carefully washed using PBS three times and subsequently analyzed on a Leica SP5 confocal microscope with a 63× oil immersion objective. Images were processed by the ImageJ software package (version 1.53o).

### 3.11. Bone Marrow-Derived Dendritic Cells (BMDCs) Culture

Bone marrow cells isolated from the femurs and tibiae of C57BL/6 mic were seeded in 12-well suspension cell culture plates (each 10^5^/mL) and incubated in an IMDM-based culture medium (5% FBS, 2 mM L-glutamine, 100 IU/mL penicillin, 100 µg/mL streptomycin and 50 µM β-mercaptoethanol; all from Sigma-Aldrich) supplemented with 10 ng/mL recombinant murine GM-CSF (R & D Systems) to obtain inflammatory BMDCs. Culture media was changed on days 2 and 6. On day 7/8, different nanoformulations were applied (1.5 µg/mL). On the following day, the samples were harvested and subjected to flow cytometric analysis or employed as stimulators in T cell proliferation assays.

### 3.12. Flow Cytometry

BMDC were harvested and transferred to FACS tubes. In order to prevent unspecific Fc-mediated binding, samples were incubated with a rat anti-mouse CD16/CD32 antibody for 10 min at 4 °C. Afterwards, BMDC were incubated with specific antibodies for DC lineage (CD11c) and activation (MHCII, CD80, CD86) markers for 20 min at 4 °C. All antibodies were obtained from BioLegend or Thermo Fisher (Waltham, MA, USA) (Table S1). Samples left untreated, incubated with a nanoformulation only, or with a single antibody served as controls. Then, the samples were washed and fixed (4% paraformaldehyde, 2 mM EDTA) and analyzed in an Attune NxT flow cytometer equipped with Attune Nxt Software v3.1.1 (both from Thermo Fisher). Data were evaluated following the gating strategy of Figure S13.

### 3.13. T-Cell Proliferation

Differentially treated BMDCs were harvested and reseeded in triplicates into 96-well plates (10^5^/mL; 100 μL/well) by serial dilution. OVA peptide-specific splenic CD4^+^ T cells from OT-II mice (https://www.ncbi.nlm.nih.gov/pubmed/9553774, accessed on 1 September 2023) were isolated via negative immunomagnetic enrichment (Miltenyi) and added as 5·10^5^/mL per 100 μL to each diluted BMDC. After one day of co-culturing, ^3^H-thymidine (0.5 μCi/well) was added for another 16–18 h of co-culture. Afterwards, cells were harvested onto glass fiber filters, and retained radioactivity was quantified in a microplate Scintillation counter (1450 MicroBeta Trilux; Perkin Elmer, Waltham, MA, USA). All samples were run in triplicate.

## 4. Conclusions

In this study, we introduced the synthesis and application of peptide-decorated polycarbonate nanogels. Starting from the controlled ROP for the synthesis of multifunctional block copolymers, polymeric micelles were assembled. Exploiting the post-polymerization modification of azide-PEG_113_-*b*-poly(MTC-PFP)_28_ at the reactive ester side chain, small molecular TLR7/8 agonists and core-crosslinkers were incorporated into these nanogels. Subsequently, the azide-moieties were shown to be presented on the surface of the nanogels and to be accessible for SPAAC via covalent conjugation of a DBCO-functional dye. Towards vaccination with peptide antigens, a CD4^+^-T-cell-specific peptide sequence of ovalbumin as a model antigen was functionalized with DBCO moieties via site-specific conjugation to its *N*-terminal cysteine. The peptide was then successfully attached to the surface of the nanocarrier to form peptide-decorated nanogels. Following in vitro studies confirmed the nanogels’ ability to effectively deliver the peptide into immune cells. Adjuvant-loading was shown to result in strong immunostimulation. Finally, the interplay of the adjuvant and peptides was demonstrated by presentation of the CD4^+^-peptide on dendritic cells, resulting in a sequence-specific and adjuvant-boosted CD4^+^ T cell proliferation. 

Consequently, azide-functional polycarbonate nanogels can be considered attractive carrier systems for both core-functionalization with small molecular cargoes and simultaneous surface decoration with peptide antigens. Our findings strongly motivate us to characterize the particles’ vaccination performance in further in vivo studies in order to address and optimize adjuvant and peptide antigen-specific immune responses. Further development of these multifunctional nanogels presented herein may pave the way for alternative peptide nanogel vaccines, especially relevant for therapeutic interventions in cancer immunotherapy.

## Data Availability

All data can be found in the manuscript or Supporting Information and can be further provided by the authors.

## Supplementary Materials

The following supporting information can be downloaded at: https://www.mdpi.com/article/10.3390/ijms242015417/s1.
