# Supplementary material for: Peptide-Decorated Degradable Polycarbonate Nanogels for Eliciting Antigen-Specific Immune Responses
Source: Int J Mol Sci. 2023 Oct 21;24(20):15417. doi: 10.3390/ijms242015417 (PMC10607756; doi:10.3390/ijms242015417)
Supplement: Supplementary file 1 [file ijms-24-15417-s001.zip › ijms-2667467-supplementary.pdf]

International Journal of
Molecular Sciences

MDPI

Supplementals

Peptide-decorated Degradable Polycarbonate Nanogels for Eliciting Antigen-Specific Immune Responses

Judith Stickdorn [1,†], Christian Czysch [1,†], Carolina Medina-Montano [2], Lara Stein[3], Lujuan Xu [1,4], Maximilian Scherger [1], Hansjörg Schild [3], Stephan Grabbe [2] and Lutz Nuhn [1,5,*]

1 Max Planck Institute for Polymer Research, 55128 Mainz, Germany
2 Department of Dermatology, University Medical Center of the Johannes Gutenberg-University Mainz, 55131 Mainz, Germany
3 Institute of Immunology, University Medical Center of the Johannes Gutenberg-University Mainz, 55131 Mainz, Germany
4 Zhejiang Cancer Hospital, The Key Laboratory of Zhejiang Province for Aptamers and Theranostics, Hangzhou Institute of Medicine (HIM), Chinese Academy of Sciences, 310022 Hangzhou, China
5 Chair for Macromolecular Chemistry, Institute of Functional Materials and Biofabrication, Department of Chemistry and Pharmacy, Julius-Maximilians-Universität Würzburg, 97074 Würzburg

† These authors contributed equally to this work.
* Correspondence: lutz.nuhn@uni-wuerzburg.de.

Citation: To be added by editorial staff during production.

Academic Editor: Firstname Lastname

Received: date
Revised: date
Accepted: date
Published: date

Copyright: © 2023 by the authors. Submitted for possible open access publication under the terms and conditions of the Creative Commons Attribution (CC BY) license (https://creativecommons.org/licenses/by/4.0/).

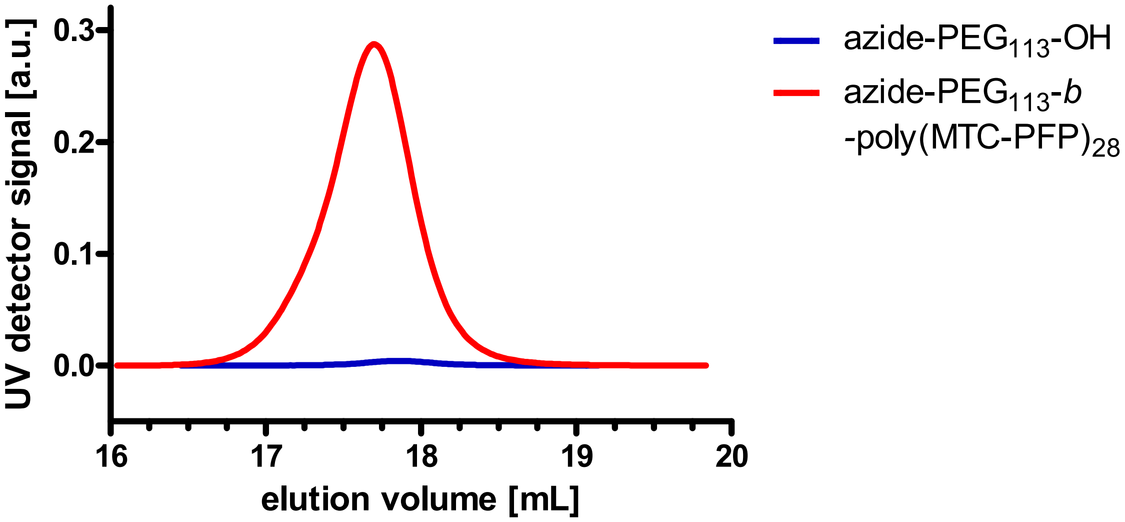

Figure S1. Size exclusion chromatography (SEC) elugram recorded by UV-Vis detector at 230 nm (UV) of azide-PEG$_{113}$-OH (blue) and azide-PEG$_{113}$-*b*-poly(MTC-PFP)$_{28}$ (red, *Đ* = 1.17) showing narrowly distributed monomodal polymers and a drastic increase of UV absorbance by polymerization of the poly(MTC-PFP) block. This indicates the synthesis of block copolymers without homopolymer impurities.

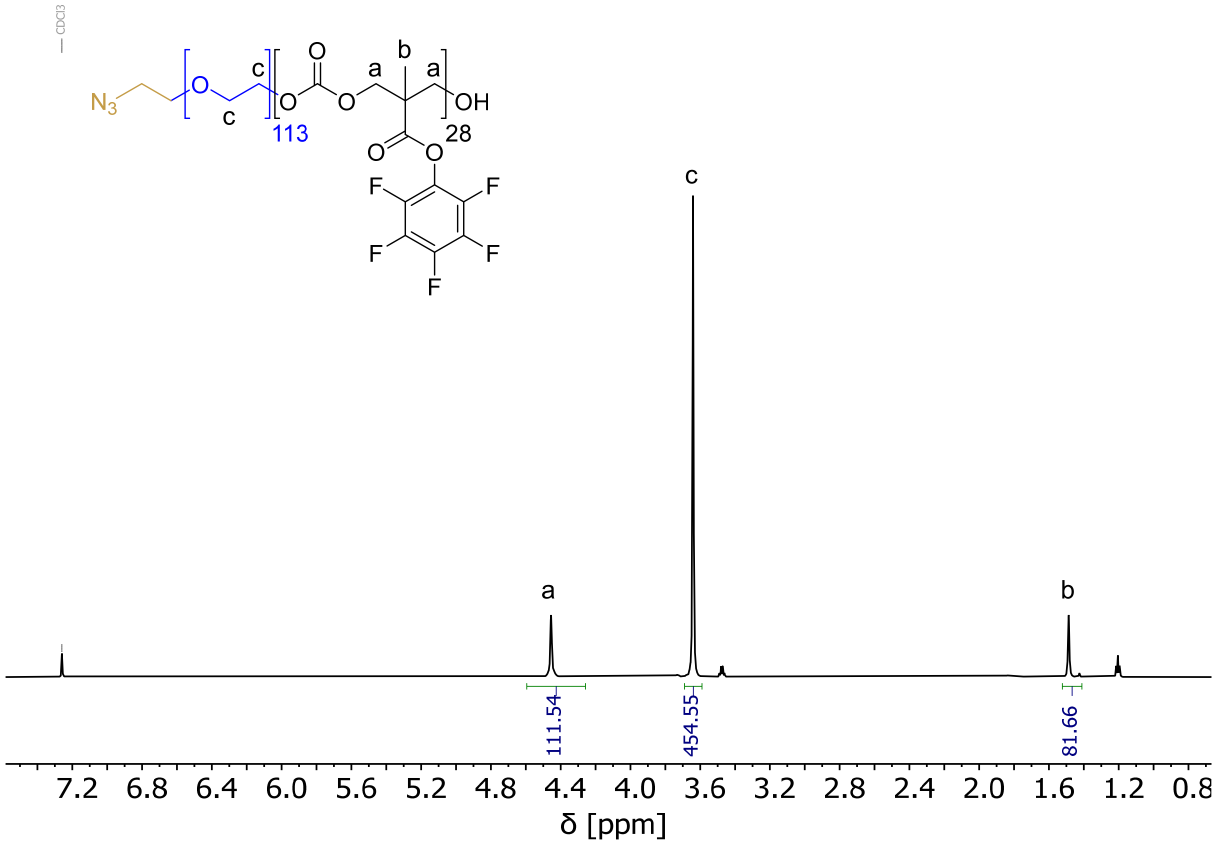

Figure S2. ^{1}H NMR spectrum of the purified block copolymer azide-PEG$_{113}$-*b*-poly(MTC-PFP)$_{28}$ dissolved in $CDCl_3$ with assignments.

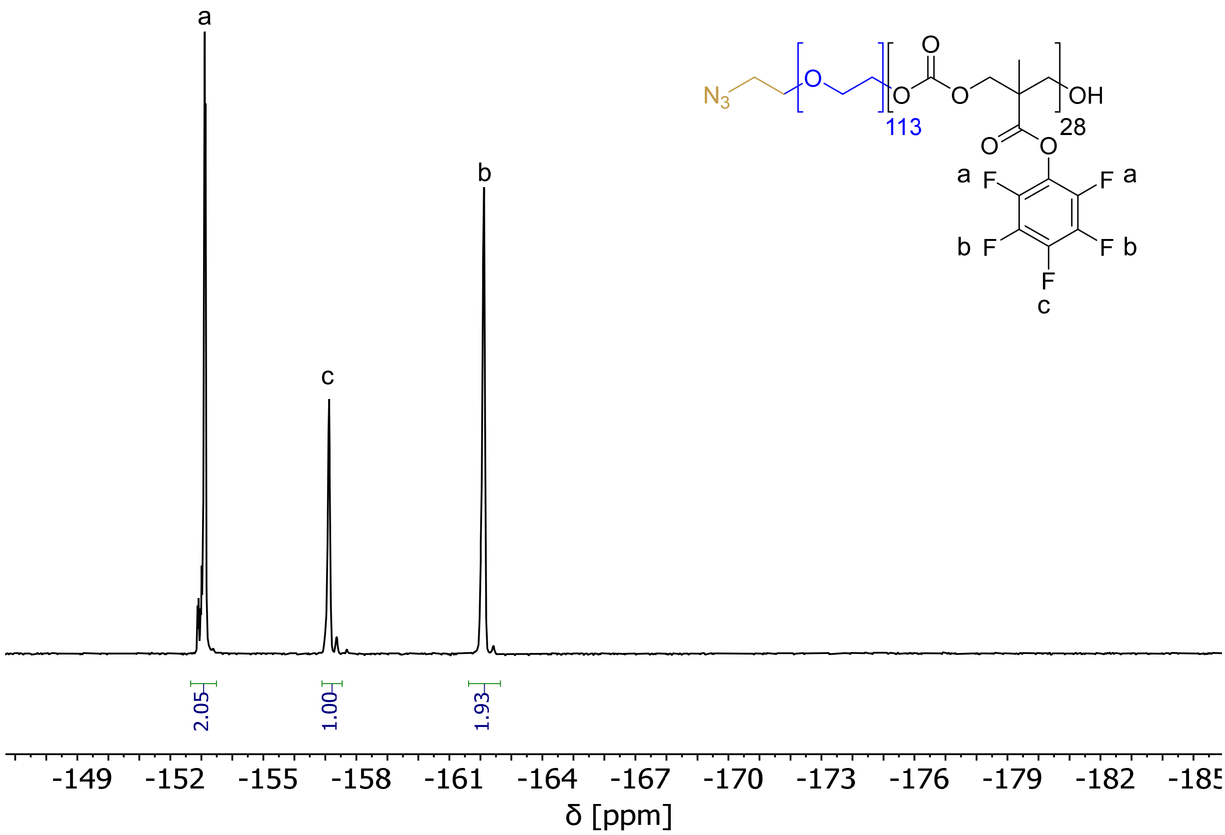

Figure S3. ^{19}F NMR spectrum of the purified block copolymer azide-PEG$_{113}$-*b*-poly(MTC-PFP)$_{28}$ dissolved in $CDCl_3$ with assignments.

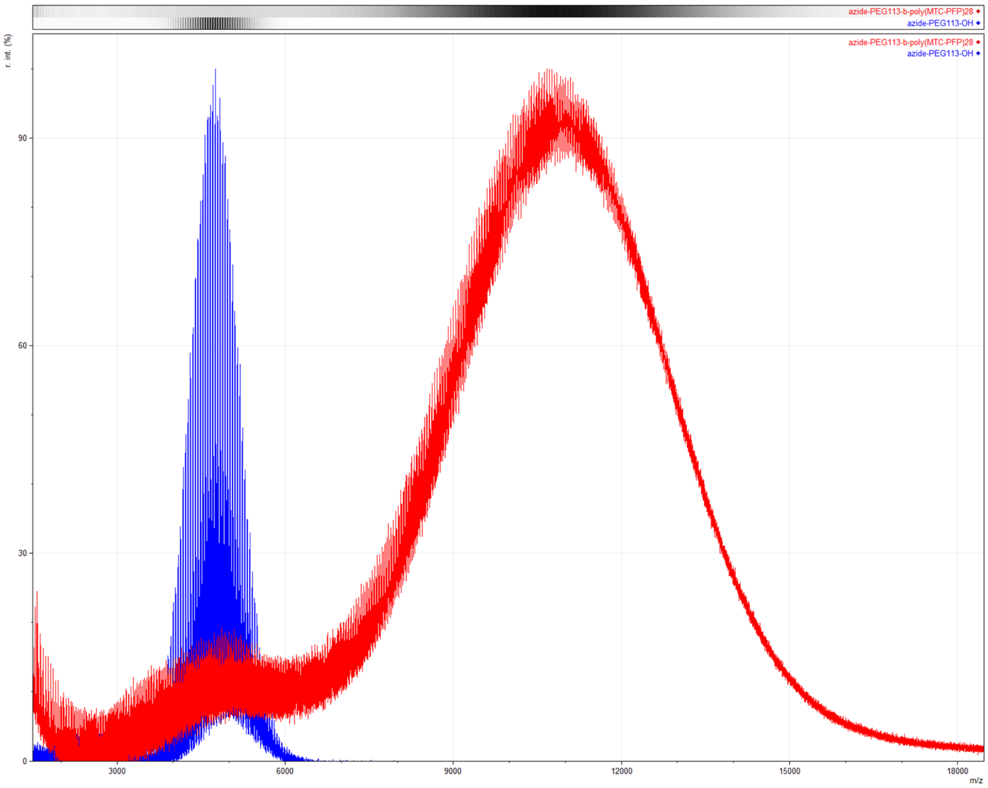

Figure S4. Mass spectrometric data (MALDI-ToF) of azide-PEG$_{113}$-OH (blue) and azide-PEG$_{113}$-*b*-poly(MTC-PFP)$_{28}$ (red) recorded in linear mode using 2-[(2E)-3-(4-tert-butylphenyl)-2-methylprop-2-enylidene]malononitrile (DCTB) as matrix.

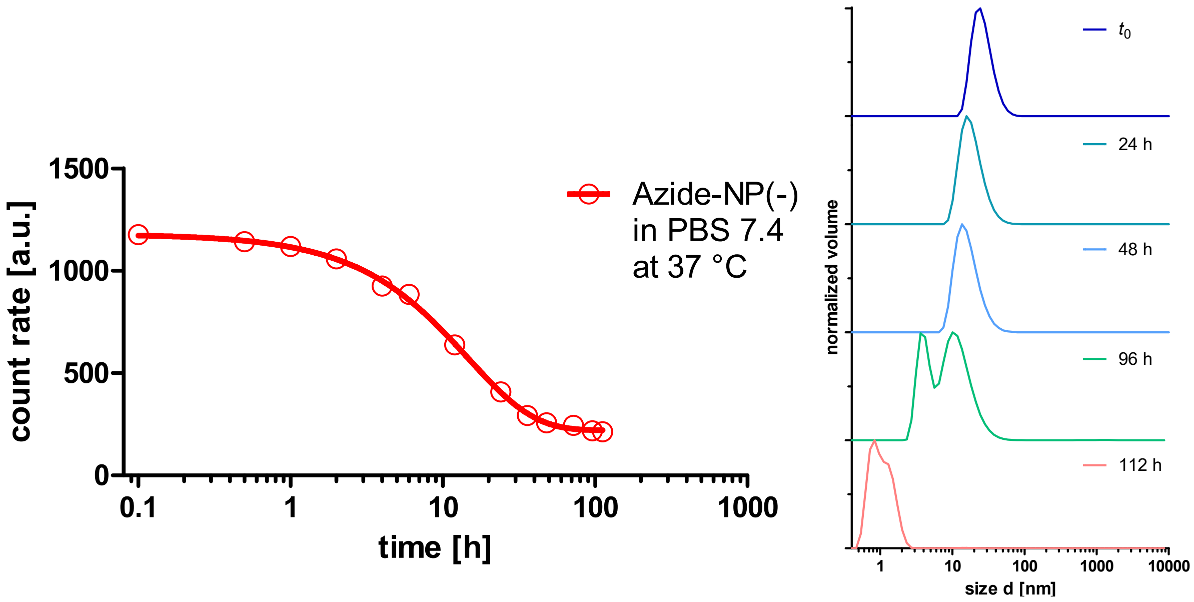

Figure S5. Degradation of azide-NP(-) in PBS (pH 7.4) at 37 °C over time investigated by DLS measurements. Dropping count rates indicate the disintegration of the particles (left graph, estimated half-life times 12-24 h). In addition to that particle sizes decrease from volume means of 27 nm at the $t = 0$ h to 18 nm after 48 h, finally reaching sizes below 5 nm after 112 h (right graph).

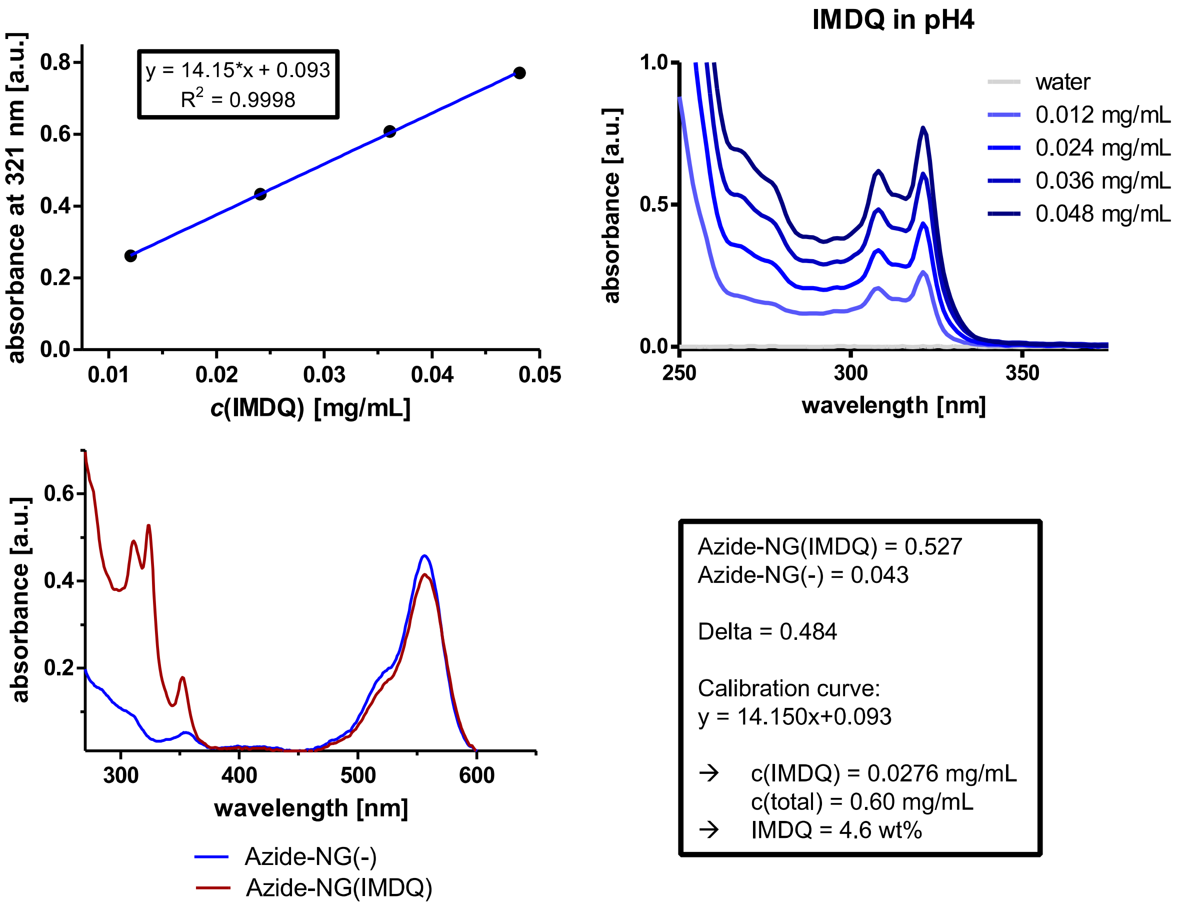

Figure S6. UV-Vis calibration of the immunodrug IMDQ in water supplemented with hydrochloric acid (pH 4) for determination of the drug-load in azide-functional nanogels by IMDQ's UV-absorbance at 321 nm. Azide-NP(IMDQ) provides an IMDQ-drug-load of 4.6 wt%. UV-Vis absorbance spectra of IMDQ in water at pH4 (right graph).

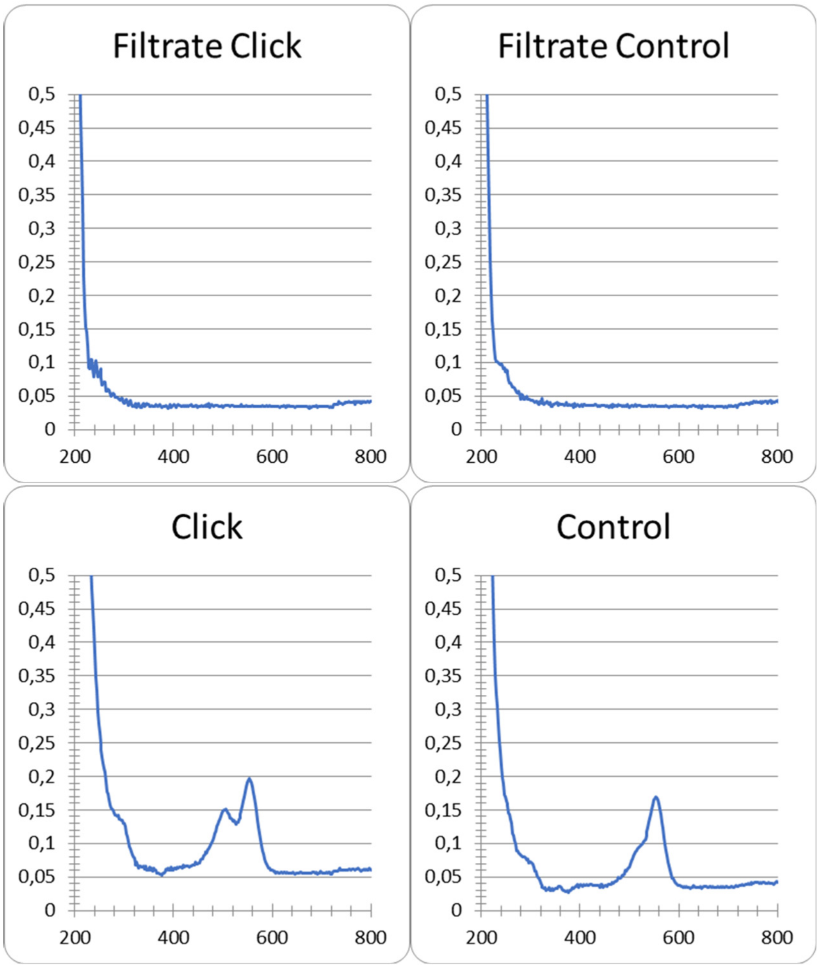

Figure S7. Additional UV-Vis spectra of the attachment of DBCO-OG488 to TMR-labeled azide-NP(-) (left spectra) and control experiment (right) of a non-SPAAC-reactive dye OG488-cadaverine to TMR-labeled azide-NP(-). Top spectra prove that after thorough spin-filtration no free (OG488) dye is present in the filtrate of both SPAAC sample and control sample, suggesting that nanogel samples were successfully purified from excess dye. The bottom spectra after SPAAC (left) shows an additional dye-absorbance of OG488 at ~488 nm. In contrast to that, no additional absorbance is found in the control sample.

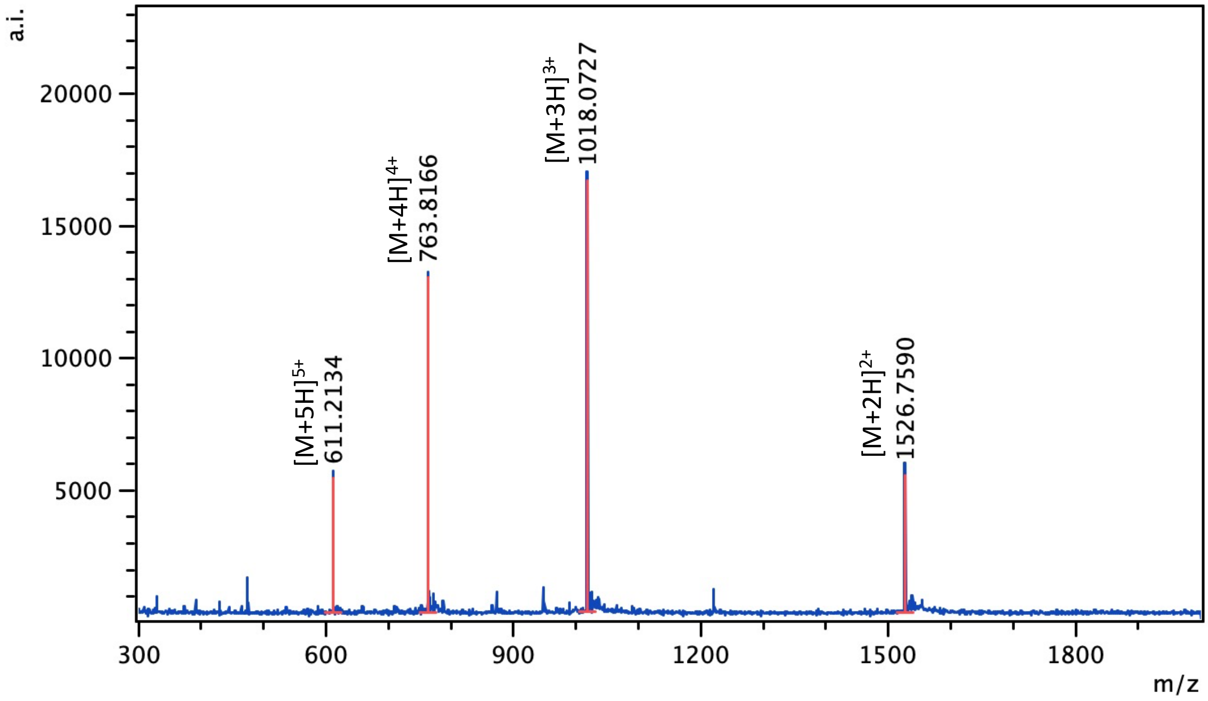

Figure S8. Mass spectrometric data of elution volume from 0-10 mL recorded by LC-MS of unmodified CD4+ epitope ionized by electro spray ionization (ESI). Peaks are assigned in the spectra and correspond to the peptide product exclusively.

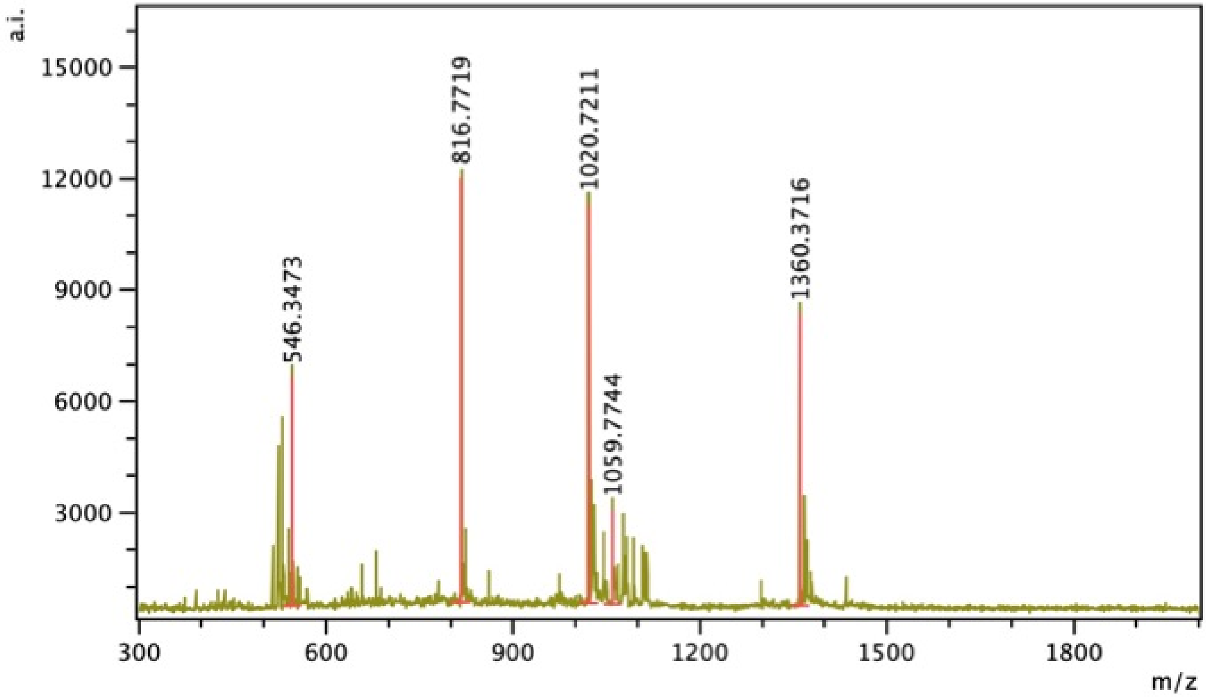

Figure S9. Mass spectrometric data of elution volume from 0-10 mL recorded by LC-MS of DBCO-modified CD4+ epitope ionized by electro spray ionization (ESI). Peaks are assigned in the spectra and correspond to the peptide product with multiple counter ions and some hydrolyzed NHS-ester DBCO-PEG12-maleimide linker.

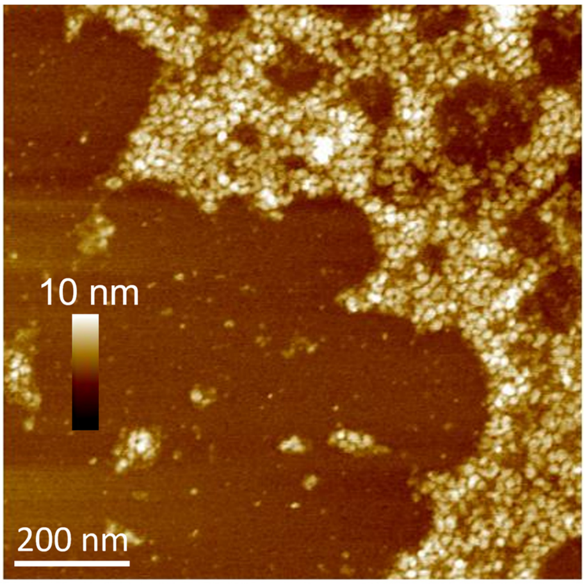

Figure S10. Atomic force microscope (AFM) image of peptide antigen decorated NP(IMDQ) showing spherical particles with heights of 5-10 nm. For sample preparation 5 µL of a 0.05 g/L particle solution in Millipore water was applied to the mica substrate (circular, 15 mm) and allowed to dry overnight.

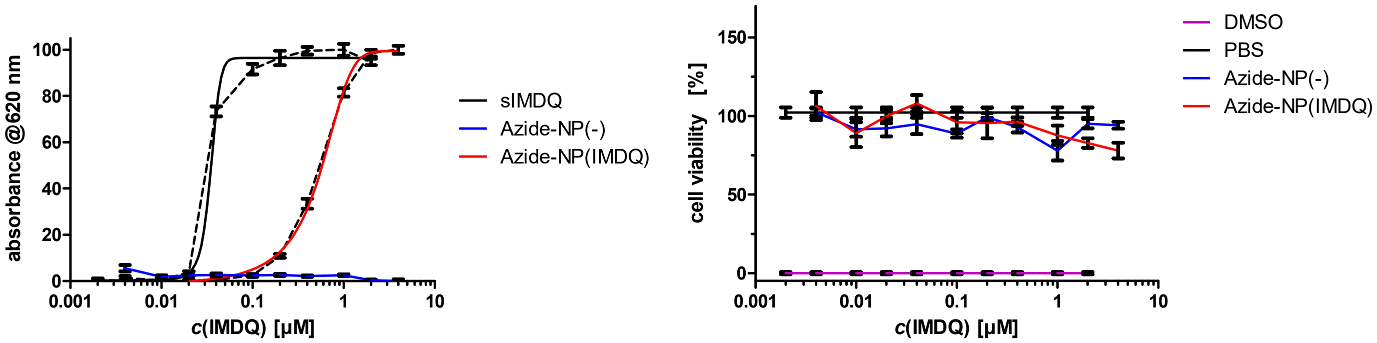

Figure S11. *In vitro* evaluation of azide-NP(-) and azide-NP(IMDQ) on RAW Blue macrophages. Adjuvant-loaded azide-NP(IMDQ) show potent immunostimulation in the range of 1 µM, whereas azide-NP(-) do not exert immunostimulating effects as determined by QuantiBlue Assay (left graph). MTT assay for cell viability confirms the high biocompatibility of azide-functional nanogels showing similar cell viability to PBS control with concentrations of IMDQ-load ranging 0.004 µM-4 µM (corresponding to particle concentrations of 0.061 µg/mL-0.061 mg/mL).

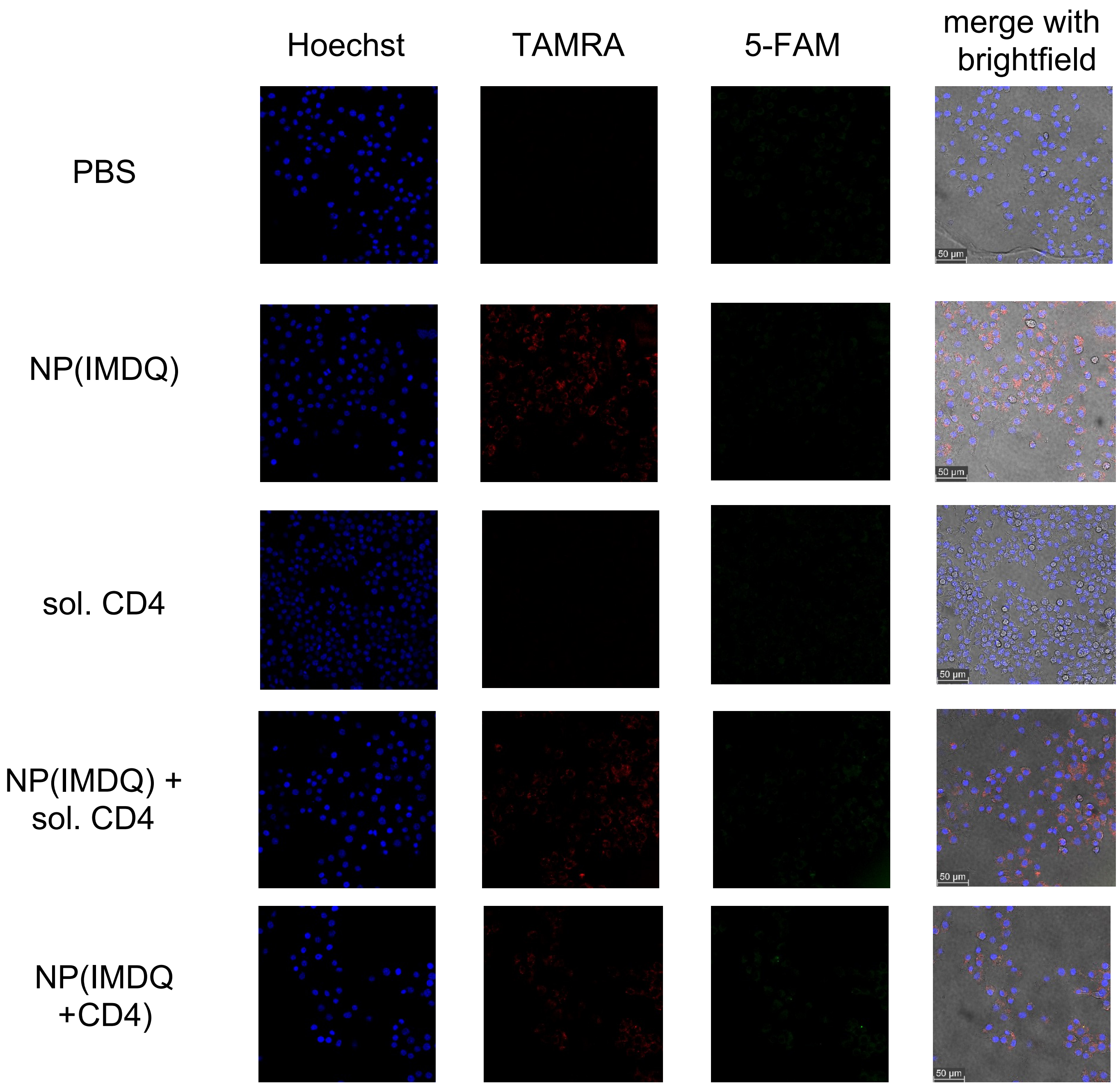

Figure S12. Additional confocal microscopy images of RAW Blue macrophages showing the internalization of nanogels by TMR fluorescence (red) and synchronously the co-uptake of CD4⁺-peptides by 5-FAM fluorescence (green) when covalently attached to the nanogel. Nuclei were stained with Hoechst dye (blue) and a bright-field images (grey) were used for visualizing cellular morphologies.

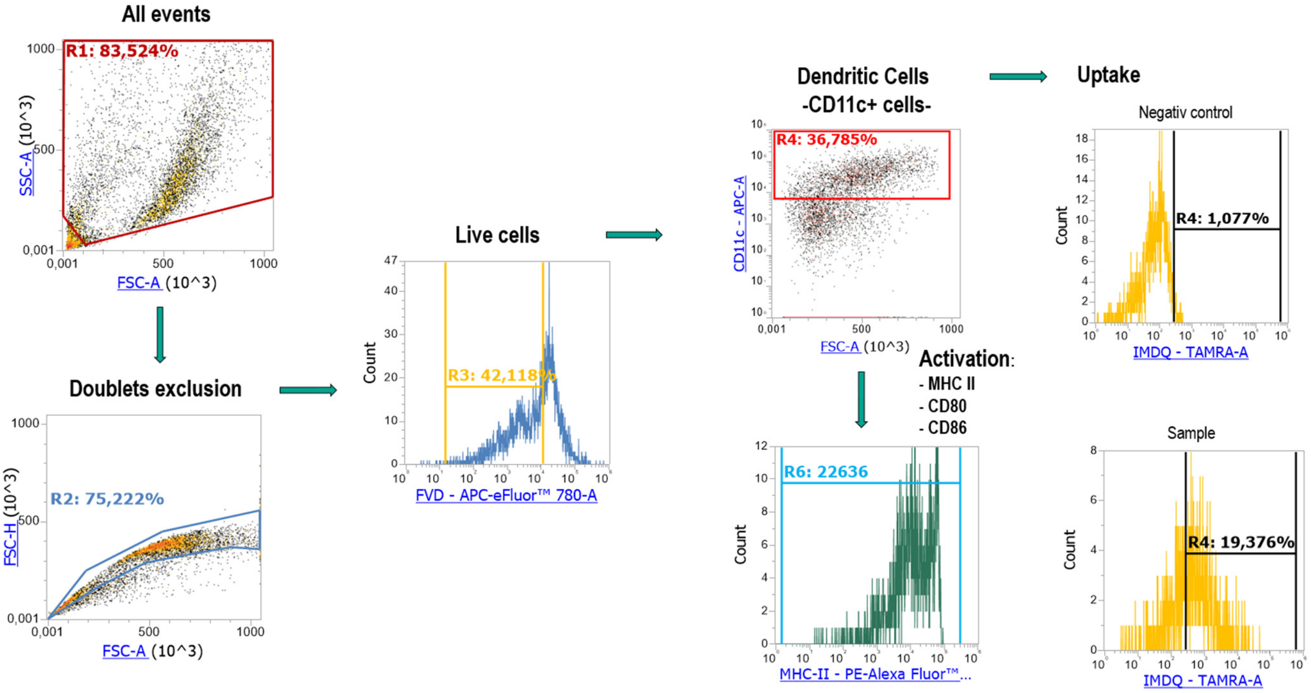

Figure S13. Gating strategy using in BMDCs (GM-CSF) to characterize dendritic cells, nanogel uptake and their activation after treatment with the different nanoparticles.

Table S1. Specific antibodies used for flow cytometry.

	Antibody	Dye
Lineage	CD11c	APC
Activation Markers	MHC-II	PE-eFl610
	CD86	eFl450
	CD80	PerCP-eFl780
Live/Dead	FVD780	eFl780
